# Reversible Cardiomyopathy in Polyarteritis Nodosa: A Case Report

**DOI:** 10.7759/cureus.83854

**Published:** 2025-05-10

**Authors:** Rafik Issa, Michael Rudy

**Affiliations:** 1 Department of Internal Medicine, University of Michigan, Ann Arbor, USA

**Keywords:** hfref, myocarditis, polyarteritis nodosa, stress cardiomyopathy, vasculitis

## Abstract

Polyarteritis nodosa (PAN) is a rare vasculitis that affects the medium-sized arteries, often involving numerous organ systems, including the heart.

In this report, we present a case of a 26-year-old male with a two-month history of systemic symptoms, abdominal pain, and intrinsic acute kidney injury, who ultimately developed acute hypoxic respiratory failure secondary to acute pulmonary edema. His echocardiogram was notable for biventricular systolic dysfunction with reduced ejection fraction and regional wall motion abnormalities, but no coronary angiogram was pursued. His cardiac magnetic resonance imaging (MRI) study did not demonstrate evidence of myocarditis per Lake Louise criteria. Renal biopsy was consistent with medium-vessel vasculitis, confirming the diagnosis of PAN. He was initiated on immunosuppression with methylprednisolone and cyclophosphamide, and subsequently placed on guideline-directed medical therapy (GDMT) with metoprolol, sacubitril-valsartan, and dapagliflozin. At one-year follow-up, his symptoms had resolved, and his ejection fraction had normalized. Our case uniquely describes a cardiomyopathy phenotype that was likely stress-mediated in the context of active inflammation and was reversible with immunosuppression and GDMT, without the need for revascularization.

## Introduction

Polyarteritis nodosa (PAN) is a systemic disorder characterized by necrotizing vasculitis of the medium-sized arteries. PAN can manifest in numerous organs, including the kidneys, gastrointestinal (GI) tract, skin, peripheral nervous system, muscles, and the heart. Cardiac involvement in PAN is clinically noted in 5%-20% of patients [1]. However, autopsies on patients with confirmed PAN demonstrate evidence of myocardial involvement in 60%-70% of cases [2]. Cardiac involvement in PAN is most commonly secondary to inflammation of the coronary arteries, with some reports suggesting involvement of the coronaries in 40%-50% of autopsies [1]. Non-ischemic heart failure with reduced ejection fraction (HFrEF) is rare and limited to a handful of case reports, where myocarditis was found to be the etiology of dysfunction [3]. In this report, we describe a rare case of reversible HFrEF, where ischemia, myocarditis, infection, metabolic, and toxin-mediated derangements were deemed unlikely culprits of dysfunction. Our case suggests that a stress-induced process, in the context of active inflammation, could be contributory to HFrEF in PAN.

## Case presentation

A 26-year-old male presented to the hospital with a history of abdominal pain, fevers, night sweats, 25-pound unintentional weight loss, and bilateral proximal interphalangeal joint (PIP) arthralgias. His symptoms first began two months prior, when he was experiencing excruciating abdominal pain. He was diagnosed with chronic cholecystitis and subsequently underwent cholecystectomy. His abdominal pain persisted, and several weeks later, he developed fever and diaphoresis. He was readmitted and started on broad-spectrum intravenous (IV) antibiotics, without resolution. Given his lack of improvement, the patient left the prior hospital and presented to our center for further management.

On arrival at our Emergency Department, his vitals were notable for a blood pressure of 169/94 mmHg, a pulse of 110 beats per minute, and an oxygen saturation of 88% on room air. He was initially afebrile but subsequently developed intermittent fevers up to 38.6 degrees Celsius. A physical exam revealed lower extremity pitting edema, mild ascites, and bilateral PIP synovitis. His labs on admission are presented in Table 1. Electrocardiogram (EKG) revealed sinus tachycardia and mildly prolonged QTc (516 ms). Computed tomography (CT) of the chest showed airspace and interstitial opacities in the lower lobes, in addition to mediastinal lymphadenopathy, while CT of the abdomen and pelvis with contrast showed bilateral hydronephrosis with no anatomical obstruction and no vascular abnormalities. Subsequent diagnostics were notable for negative blood cultures, viral studies, urine drug screen, rheumatologic serologies, and anti-neutrophil cytoplasmic antibody (ANCA) panels. His initial transthoracic echocardiogram was reassuring, with a left ventricular ejection fraction (LVEF) of 55% and no wall motion abnormalities.

**Table 1:** Laboratory values on admission

Lab	Value	Reference Range
White Blood Cell Count	21.7 K/µL	4-10 K/µL
Hemoglobin	8.3 g/dL	13.5-17.0 g/dL
Platelets	566 K/µL	150-400 K/µL
Blood Urea Nitrogen	14 mg/dL	8-20 mg/dL
Creatinine	1.71 mg/dL	0.70-1.3 mg/dL
Aspartate Aminotransferase	67 U/L	<34 U/L
Alanine Aminotransferase	26 U/L	10-49 U/L
Alkaline Phosphate	152 U/L	40-116 U/L
Brain Naturietic Peptide	1327 pg/mL	<100 pg/mL
High Sensitivity Troponin (initial)	95 pg/mL	0-19 pg/mL
High Sensitivity Troponin (2 hours)	97 pg/mL	0-19 pg/mL
Erythrocyte Sedimentation Rate	126 mm	0-15 mm
C-Reactive Protein	21.2 mg/dL	<0.6 mg/dL

**Figure 1:**
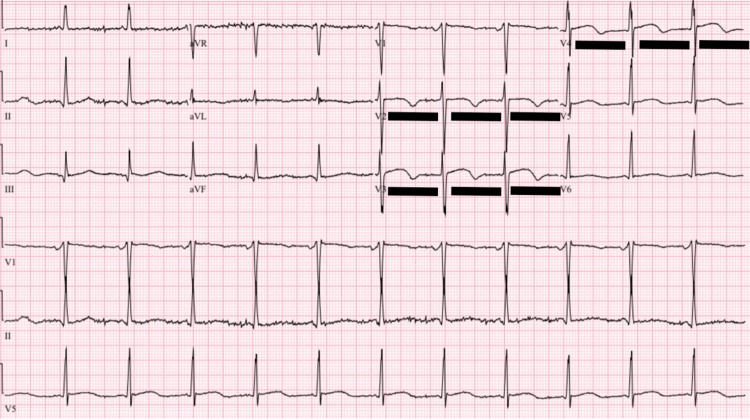
EKG at the time of respiratory decompensation This EKG demonstrates normal sinus rhythm, with biphasic T-wave inversions in V2-V4 (underlined in black). This pattern could be suggestive of ischemia in the anterior wall of the left ventricle. EKG, electrocardiogram

**Figure 2:**
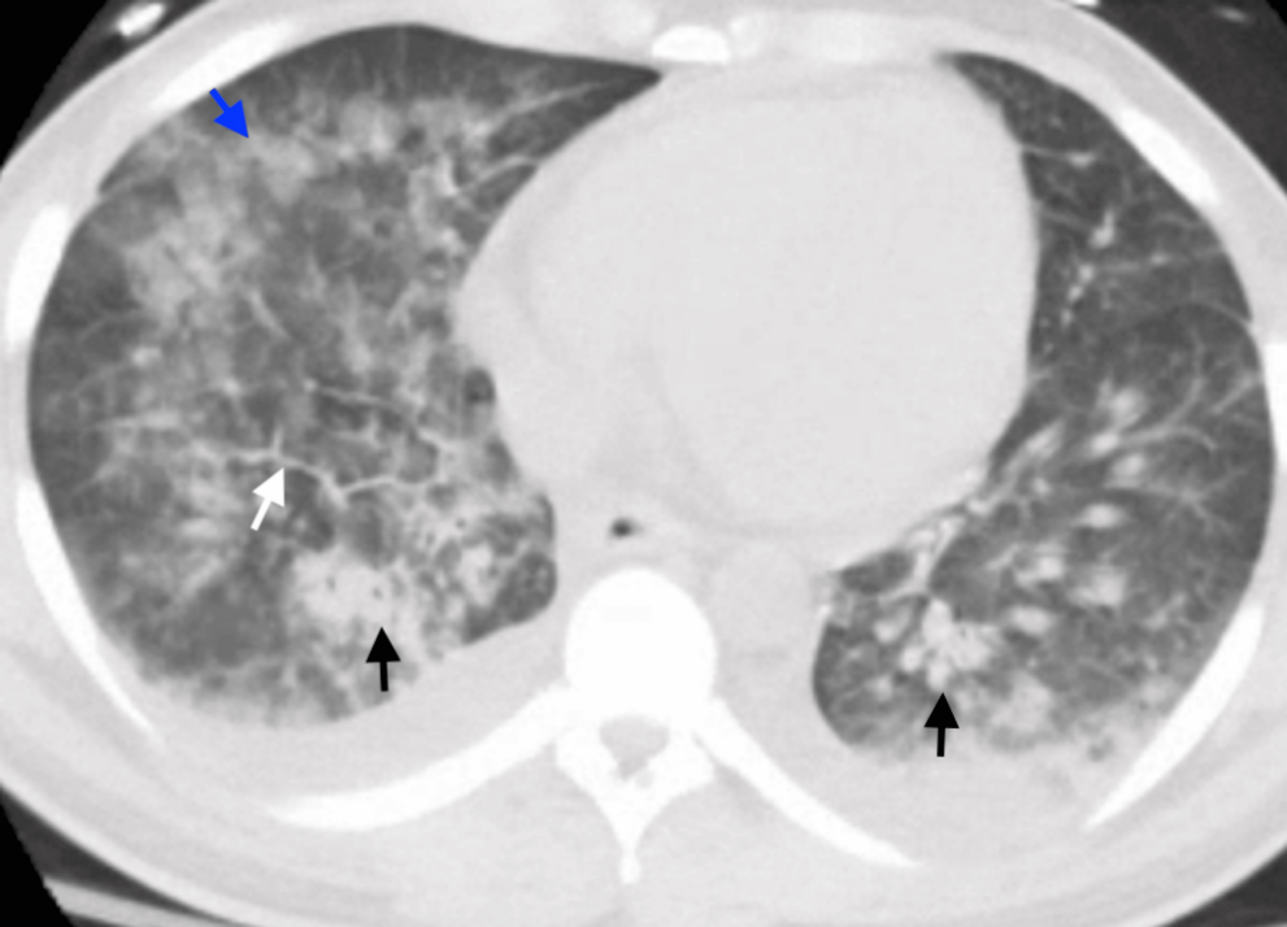
CT chest with contrast at time of respiratory decompensation This CT scan is notable for bilateral airspace consolidative opacities (black arrows), ground-glass opacities (blue arrow), and interlobular septal thickening (white arrow). These findings are most suggestive of pulmonary edema, but could also be seen with diffuse alveolar hemorrhage in the right clinical context. CT, computed tomography

Two days following his presentation to the hospital, the patient began coughing blood-tinged sputum, became hypertensive to 180/100, and developed increasing oxygen requirements, ultimately requiring intubation. His repeat evaluation was notable for high-sensitivity troponin levels of 147, 207, and 217 before down-trending, and a brain natriuretic peptide of 3211 pg/µL. EKG (Figure 1) now demonstrated biphasic T-wave inversions in V2-V4, and CT chest (Figure 2) revealed worsening airspace opacities concerning for pulmonary edema versus diffuse alveolar hemorrhage (DAH). A repeat echocardiogram was performed three days after initial presentation and was concerning for biventricular failure, with an LVEF of 37% and regional akinesis in the inferior, inferolateral, and anterolateral walls. A bronchoscopic alveolar lavage was performed on the day of intubation, without hemorrhagic return and showing an 85% neutrophilic-predominant cell differential, making DAH less likely. The patient’s hypoxia persisted despite blood pressure control with a nicardipine infusion. He was diuresed with IV furosemide. Due to multisystemic involvement and failure to improve on antibiotics during his prior admission, an autoimmune process was suspected, and he was empirically started on pulse-dose IV methylprednisolone 1000 mg for three days before transitioning to prednisone 60 mg. With these interventions, the patient’s hypoxemia resolved, and he was extubated within three days.

Given the elusive underlying etiology, a tissue diagnosis was pursued through a renal biopsy. The biopsy demonstrated severe medium-vessel arteritis, mixed fibrinoid necrosis, and evolving luminal fibrosis, consistent with an active subacute process. There was minimal glomerular involvement with rare red blood cell casts, making glomerulonephritis unlikely. There was no evidence of granulomas, giant cells, or eosinophils. These findings, in conjunction with his clinical presentation, were felt to be most consistent with a diagnosis of PAN.

The patient was continued on a prednisone taper and treated with IV cyclophosphamide. In the outpatient setting, he completed the cyclophosphamide course and was later maintained on methotrexate. From a cardiac standpoint, a follow-up cardiac magnetic resonance imaging (MRI) study in three months showed persistently reduced LVEF of 39% with normalized right ventricular (RV) systolic function (Video 1), consistent with delayed myocardial recovery. Neither late gadolinium enhancement nor increased T2 signal was observed; given the absence of necrosis, hyperemia, and edema, he did not meet Lake Louise criteria for myocarditis on imaging. He was initiated on guideline-directed medical therapy (GDMT) with sacubitril-valsartan, metoprolol succinate, and dapagliflozin. A repeat echocardiogram in one year remarkably showed a normalized LVEF. Inflammatory markers were not tracked. At this stage, the patient had returned to baseline function and was asymptomatic. Of note, a cardiac catheterization was not pursued, given patient improvement. A coronary CT angiography was ordered, but the patient did not follow up with that test.

**Video 1:** Cardiac MRI at three-month follow-up Cardiac MRI was obtained three months following his hospital presentation. The video above depicts cine-sequence imaging. The sequence reveals left ventricular dilatation and a moderately reduced LVEF (~39%). MRI, magnetic resonance imaging; LVEF, left ventricular ejection fraction

## Discussion

In this report, we present a case of a patient who had multisystemic symptoms, was found to have PAN on renal biopsy, and whose course was complicated by HFrEF. Renal disease affects as much as 50% of patients with PAN; the pathophysiology centers around partial luminal stenosis of the renal vasculature, which can lead to ischemia of the glomeruli without frank glomerulonephritis [4]. Cardiac involvement in PAN is predictive of increased mortality and morbidity [5]. Broadly, the cardiac manifestations of PAN can be classified into four categories.

First, arteritis of the major epicardial coronaries is seen in 40%-50% of cases [1]. The inflammation can subsequently result in coronary occlusions and aneurysmal formation, leading to acute coronary syndromes (ACS) [6-10]. A subset of patients develops arteritis of the small myocardial arterioles without epicardial coronary involvement [11]. Evidence of myocardial infarction (MI) on autopsy reports ranges from 11% to 62%, although a much smaller subset of patients develops clinically apparent ACS [2]. Furthermore, some cases are suggestive of acute MI secondary to vasospasm [12]. Interestingly, coronary artery disease (CAD) in some patients can progress even when the disease appears otherwise inactive based on other inflammatory markers [10]. The angiographic findings of CAD development seem to be independent of clinical findings, making it difficult to monitor progression until a patient presents with symptomatic angina or ACS. Prospective studies are needed to study the best strategies for follow-up in quiescent PAN. Coronary vasculitis is treated with immunosuppression and revascularization as appropriate, with percutaneous coronary intervention (PCI) for single-vessel stenosis, endovascular coiling for aneurysms, or coronary artery bypass grafting (CABG) when the disease appears diffuse and distal targets are viable for graft anastomosis [8,13].

Second, hypertension is a major driver of heart failure in PAN [11]. PAN can attack the renal artery, alter renal blood flow, and activate the renin-angiotensin-aldosterone system (RAAS), leading to secondary hypertension. Uncontrolled hypertension can thereby result in left ventricular hypertrophy and diastolic dysfunction. Involvement of the renal arteries was not observed in our patient. Third, non-uremic pericarditis has been described in 5%-25% of patients [14]. Fourth, myocarditis is possible. Myocarditis is uncommon in PAN and was primarily noted on autopsy reports, although recent case reports have highlighted evidence of in vivo myocarditis without coronary vasculitis [3].

PAN with cardiac involvement is classified as severe and is thus treated with steroids and cyclophosphamide induction. Once remission has been achieved, various steroid-sparing agents, including methotrexate, azathioprine, or mycophenolate, are utilized [15].

In this patient, our patient’s acute respiratory decompensation was most likely due to acute pulmonary edema and an inflammatory reaction affecting his heart and lungs. This is in line with his response to diuretics, antihypertensives, and corticosteroids. The patient’s troponin elevation was most likely secondary to demand and inflammation rather than primary ACS, triggering decompensation. The etiology of the patient’s reduced ejection fraction is more intriguing. Possibilities include ischemic cardiomyopathy, given the focal wall motion abnormalities, but his LVEF improved without revascularization, and he had no chest pain. However, without coronary angiography, we cannot rule out this possibility with certainty. Myocarditis is another possibility, especially in the context of elevated inflammatory markers, but the cardiac MRI did not show evidence of this. The sensitivity of cardiac MRI can be as high as 92%, but it is possible that the patient may have had myocarditis that could have been seen on an endomyocardial biopsy [16]. Hypertensive cardiomyopathy could not explain the acute onset of his cardiac dysfunction. Laboratory work-up had excluded major infectious and toxin-mediated causes of systolic dysfunction. As such, an inflammatory stress-mediated cardiomyopathy in the setting of active PAN and elevated inflammatory markers that is responsive to immunosuppression was suspected to be the most likely etiology of his decompensation.

Few cases in the literature have documented HFrEF that has recovered in the setting of PAN. Among those cases, the patients who recovered their systolic function either had evidence of CAD that was revascularized or evidence of myocarditis that was treated with immunosuppression [3]. Thus, our case presents a phenotype of likely non-ischemic PAN-cardiomyopathy that is reversible despite the absence of signs of myocarditis on imaging.

## Conclusions

Cardiac involvement in PAN has serious implications for patient outcomes. PAN-associated cardiomyopathy is characterized by various phenotypes, each of which requires a tailored management approach. Although the damage from PAN can be permanent, certain forms of PAN cardiomyopathy are reversible when recognized and treated appropriately. Here, we describe a form of PAN cardiomyopathy that is most akin to stress cardiomyopathy in the setting of active inflammation. Providers should be aware of this entity, as immunosuppression and GDMT could restore normal systolic function, even when patients lack findings of myocarditis on imaging or CAD. Our case is limited by the absence of coronary angiography and endomyocardial biopsy findings to confirm the exclusion of CAD and myocarditis, respectively. Future prospective studies focusing on the trajectory and outcomes of patients with PAN-cardiomyopathy can help in delineating the various phenotypes and their appropriate treatment strategies.
